# Brazos Valley Medical Association

**Published:** 1898-11

**Authors:** 


### BRAZOS VALLEY MEDICAL ASSOCIATION.

### Preliminary Announcement and Program.

BRYAN, TEXAS, October 15, 1898.

DEAR DOCTOR: I present herewith the announcement of the Sixth Semi-Annual Meeting of the Brazos Valley Medical Association, which convenes at Rockdale, Texas, on Tuesday and Wednesday, the fifteenth and sixteenth of November, 1898.

The programme is exceedingly interesting and will be entertaining as well as instructive. Positive arrangements have been made for the exhibition of X-rays, which will take place the first day of the meeting. This in itself will be sufficiently instructive to repay you for your visit. The members are invited to contribute subjects for examination.

The last afternoon will be devoted to clinical work.

In addition to the above interesting features, we will have an address on "Medical Jurisprudence." by Hon. T. S. Henderson, Regent of the University of Texas.

The local physicians are making extensive arrangements to entertain us, and let us show our appreciation by attending.

This promises to be the most successful meeting in the history of the Association and I hope to see *every* member present. Come, you will not regret it. I am yours fraternally,

GEORGE R. TABOR, M. D.,
President.

PROGRAMME.

X-Ray Illustrations and Examinations.—By R. S. Hyer, Professor of Natural Science, Southwestern University, Georgetown.

Address on "Medical Jurisprudence."—Hon. T. S. Henderson, Regent of the University of Texas, Cameron.

### SECTIONS.

SECTION ON OBSTETRICS AND DISEASES OF CHILDREN.

1. Report of Chairman.—Dr. H. W. Cummings, Hearne.

2. Paper.—"A Difficult Labor." Dr. R. S. Wallis, Rockdale. Discussion by Dr. T. A. Pope, Cameron.

3. Paper.—"Pregnancy, and Some Troubles Incidental Thereto." Dr. T. L. Todd, Harvey. Discussion by Dr. Daniel Parker, Calvert.

4. Paper.—"Eclampsia, Gravidarum et Parturientium." Dr.

M. L. Langford, Baileyville. Discussion by Dr. G. W. Emory, Anderson.

5. Paper.—"Infant Mortality During Labor, and Its Prevention." Dr. George F. Hulbert, President Woman's Hospital, St. Louis. Discussion by Dr. P. M. Raysor, Bryan.

6. Paper.—"Indigestion in Children." Dr. E. S. Ferguson, Cameron. Discussion by Dr. A. J. Ellzy, Lilac.

7. Paper.—"Infantile Diarrhœa"—Report of Cases. Dr. E. A. Thompson, Navasota. Discussion of causes, prevention and treatment open to Association.

Dr. E. A. Thompson, Navasota, Secretary of Section.

SECTION ON GENERAL MEDICINE.

1. Report of Chairman.—Dr. G. M. Abney, Franklin. With paper on "Chronic Malarial Poison—Some of its Results and Treatment." Discussion by Dr. J. M. Nicks, Stone City.

2. Paper.—"Remittent Fevers." Dr. O. T. Lewis, Millican. Discussion by Dr. G. C. McLeod, Buckholts.

3. Paper.—"Pernicious Malarial Fevers." Dr. W. P. Gilstrap, Wheelock. Discussion by Dr. J. P. Oliver, Caldwell.

4. Paper.—"Pneumonia." Dr. R. W. Wallis, Rockdale. Discussion by Dr. S. J. Emory, Navasota.

5. Paper.—"Typhoid Fever, Diagnosis and Treatment." Dr. J. C. Holman, Franklin. Discussion by Dr. W. F. Sharp, Davilla.

6. Paper.—"Neurosis of the Stomach." Dr. J. A. Boyd, Thorndale. Discussion by Dr. L. M. Bassett, Hearne.

7. Paper.—"Complete Paralysis—Sensation and Motion—Due to Malarial Toxæmia—Recovery.—Report of Case." Dr. F. R. Collard, Wheelock. Discussion by Dr. J. M. Dollar, Gause.

Dr. J. A. Boyd, Thorndale, Secretary of Section.

SECTION ON SURGERY AND GYNAECOLOGY.

1. Report of Chairman.—Dr. E. A. Harris, Navasota. With paper on the "Normal Saline Solution." Discussion by Dr. I. P. Sessions, Rockdale.

2. Paper.—"Bites of Rabid Animals—Report of Cases." Dr. W. B. Briggs, Easterly. Discussion by Dr. R. K. Fountain, Jones Prairie.

3. Paper.—"End to End Intestinal Anastomosis." Dr. W. W. Greer, Cameron. Discussion by Dr. A. C. Scott, Temple.

4. Paper.—"A New Method of Intestinal Anastomosis." Dr. J. F. Eaves, Millican. Discussion by Dr. H. L. Fountain, Bryan.

5. Paper.—"Gonorrhœa in the Female." Dr. F. Johnson, Anderson. Discussion by Dr. J. W. Hudson, Milano.

6. Paper.—"Appendicitis—Report of Cases." Dr. T. L. Treadaway, Lilac. Discussion by Drs. W. W. Greer and E. S. Ferguson, Cameron.

Dr. J. F. Eaves, Millican, Secretary of Section.

Report of Cases on Eye, Ear, Throat and Nose.—Dr. E. P. Daviss, Houston; Dr. J. M. Woodson, Temple.

The members are requested to report cases having come under their observation.

